# Detection and Imaging of Damages and Defects in Fibre-Reinforced Composites by Magnetic Resonance Technique

**DOI:** 10.3390/ma14040977

**Published:** 2021-02-19

**Authors:** Carine L. Alves, Janete S. Oliveira, Alberto Tannus, Alessandra Cristina Soares P. Tarpani, José R. Tarpani

**Affiliations:** 1 Materials Engineering Department, Engineering School of São Carlos, University of São Paulo, São Carlos, SP 13590-566, Brazil; carine.lourenco.alves@tuhh.de (C.L.A.); janeteschultz@yahoo.com.br (J.S.O.); alessandra.tarpani@usp.br (A.C.S.P.T.); 2 Physics and Informatics Department, Physics Institute of São Carlos, University of São Paulo, São Carlos, SP 13590-566, Brazil; goiano@ifsc.usp.br

**Keywords:** damage and defect assessment, magnetic resonance imaging, permanent implant material, polymer matrix composite

## Abstract

Defectively manufactured and deliberately damaged composite laminates fabricated with different continuous reinforcing fibres (respectively, carbon and glass) and polymer matrices (respectively, thermoset and thermoplastic) were inspected in magnetic resonance imaging equipment. Two pulse sequences were evaluated during non-destructive examination conducted in saline solution-immersed samples to simulate load-bearing orthopaedic implants permanently in contact with biofluids. The orientation, positioning, shape, and especially the size of translaminar and delamination fractures were determined according to stringent structural assessment criteria. The spatial distribution, shape, and contours of water-filled voids were sufficiently delineated to infer the amount of absorbed water if thinner image slices than this study were used. The surface texture of composite specimens featuring roughness, waviness, indentation, crushing, and scratches was outlined, with fortuitous artefacts not impairing the image quality and interpretation. Low electromagnetic shielding glass fibres delivered the highest, while electrically conductive carbon fibres produced the poorest quality images, particularly when blended with thermoplastic polymer, though reliable image interpretation was still attainable.

## 1. Introduction

Compared to structural metallic alloys, high-performance fibre-reinforced polymer (FRP) composites exhibit low density, high specific stiffness and strength (per unit mass), corrosion resistance and remarkable design flexibility. They can operate under different mechanical, thermal and environmental conditions, so that they are employed in several engineering fields [1].

Specifically, in regard to load-bearing surgically implanted orthopaedic prostheses, a medical field where FRPs are increasingly being used, the life expectancy growth of the human population and the number of victims of traumatic incidents has boosted the need for advanced materials for structural implants. Thermoset and thermoplastic matrix FRP composites have been widely accepted due to their exceptional versatility for permanently and temporarily implanted orthopaedic devices [2,3,4,5,6]. The biocompatibility of FRP also provides support to the appropriate cellular activity, assisting the regeneration of soft and hard tissues [7,8,9,10].

Human body implants have a finite lifespan, typically following long-term use, i.e., fatigue, in continuous contact with biofluids. However, implants can be damaged and thus, can exhibit pre-mature failure which reduces the expected in-service life [11,12,13,14,15,16,17].

Non-destructive, non-invasive, painless, reliable, non-lethal radiation, fast and high penetrating power examination techniques for determining the degree of structural integrity of advanced FRP orthopaedic implants are therefore highly desirable.

Magnetic resonance imaging (MRI) [18,19,20,21] has a number of important advantages with respect to traditional concurrent techniques, due to its non-health-threatening nature, against X-ray radiography and eddy current [22], independence from damage orientation, in contrast to ultrasonics [23], a high penetration power of electromagnetic field, differently from the surface-confined analysis provided by lock-in infrared thermography [24], besides unrestricted use with electrically (carbon fibre—CF) and non-electrically (glass fibre—GF) conductive FRP composites.

Moreover, since biofluids are proton-rich compounds and quite sensitive to the magnetic resonance effect of an external magnetic field and atomic nuclei interaction, the MRI technique is naturally a very promising tool for the structural integrity assessment of FRP implants [25].

Though the medical literature provides some in vivo magnetic resonance images of implanted FRP orthopaedic devices [26,27,28,29], none of the articles addressed the potentialities of MRI to detect and characterize manufacturing defects and in-service damages.

Longo and Koeneman [29] predicted that radiography, to date the most utilized technique for the non-destructive examination of conventional metallic implanted orthopaedic prostheses [30], would become a powerful in vitro and/or in vivo technique to detect and characterize minor changes of the structural integrity of complex composite implanted prostheses; however, the state of art has shown that this goal has not been achieved, despite the huge potential offered by X-rays to monitor with unbeatable accuracy the damage initiation and progression in this class of materials under dry environments [31,32].

The most recent attempt in this regard has been conducted by Vercio and Basmajian [33], who succeeded in identifying the gross failure of a carbon fibre-reinforced intramedullary nail, but did not prosper in characterizing the subtle discontinuities which could have led to the fracture event, due to the lack of sufficient image contrast to detect minor cracks and defects in the target device. The increasing risk of long-term effects of ionizing radiation, such as cancer, along with the expensive use of contrast enhancer gold coating incorporated into the composite material [34] and the need for a potentially allergenic intravenous iodinated radio-contrasting agent to overcome the typical radiolucency of polymer matrices [35], will most likely counteract the near-term implementation of X-ray techniques in this non-invasive procedure for permanent polymer-based structural implants [32].

Ultrasonic-based methods have also attracted great interest towards the structural integrity assessment of permanent medical implants, e.g., elastic-guided waves [36], however, promising results have been shown to apply only to the monitoring of prosthetics’ osseointegration along the rehabilitation process.

Relying on the author’s experience and expertise built during previous MRI research in composite structures [37], the present study evaluates the potential of multi-slice MRI techniques for the in vitro non-destructive inspection of defective and damaged FRP specimens immersed in simulated biofluid.

The rationale is that damages and defects are either percolated or filled with the proton-rich liquid, so that they can be promptly imaged since the magnetic resonance-sensitive biofluid performs as an intrinsic contrast agent.

The obtained results are expected to contribute to the development and implementation of in vivo non-health-threatening multi-examination procedures to determine the severity and criticality levels of manufacturing defects and in-service damages in FRP orthopaedic implants. This may possibly postpone or even avoid surgeries to replace them, i.e., in-service lifetime extension, largely benefiting the patient’s well-being. In cases where medical procedures are inevitable, an MRI could also provide surgeon guidance towards the best surgical strategy.

## 2. Composite Laminates and Test Coupons

Non-destructively examined composite laminates, and the specimens’ preparation procedures are described below.

### 2.1. FRP Laminates

Three different FRP systems were evaluated in this study, as follows:

A 5 mm-thick laminate comprising continuous carbon fibre-reinforced epoxy resin (EPX-CF) manufactured at the industrial scale via vacuum bag autoclave processing 24 layers of 0/90 bidirectional plain-weave fibre fabric (Hexcel™, 193 g/m^2^ areal weight), pre-impregnated with an EPX system (Araldite LY 5052 resin and Aradur 5052 polyamines mixture also supplied by Hexcel™) piled up to [(0/90)/± 45_2_/(0/90)]_6_ architecture and subsequently cured at 180 °C. The fibre volume fraction targeted by the composite manufacturer was 60%.

A 5 mm-thick laminate comprising continuous carbon fibre-reinforced thermoplastic poly-phenylene sulphide (PPS-CF) also fabricated at the industrial scale via the hot compressing (300 °C) 16 layers of 0/90 bidirectional 5-harness satin (5HS) CF fabric, semi-impregnated with PPS (Tencate™, 280 g/m^2^ areal weight) stacked to [(0/90)/± 45_2_/(0/90)]_4_ array. The composite producer established the proper fibre volume fraction as 50%.

A 3.5 mm-thick laminate comprising continuous glass fibre-reinforced thermoset epoxy resin (EPX-GF) produced at the laboratory scale by vacuum-assisted infusion on the flexible tooling 20 layers of 0/90 bidirectional plain-weave dry fibre fabric (Fibertex-Brazil™, 200 g/m^2^ areal weight), stacked to [0/90]_10S_ array in liquid EPX resin system (Araldite LY 1316-2BR resin, HY 1316 hardener and Aradur 2954 curing agent provided by Huntsman™-Brazil) and later cured at 80 °C. Typically, this fabrication technique results in fibre-to-resin ratios of around 50:50.

In regard to the materials tested, it is worth mentioning that though some restrictions to EPX-CF composites in biomedical applications still persist, due to concerns about leachable toxic rest monomers [38,39,40], carbon and glass FRP implants are still in service [41,42] and remain a matter of interest for in-vivo non-destructive examination. On the other hand, PPS-CF and similar sulphonated thermoplastic composites have already been utilized as prosthetic implants, due to their superior mechanical properties, cytocompatibility and in vivo osteogenesis [2,4].

### 2.2. Purposely Damaged Test Specimens

**Figure 1:**
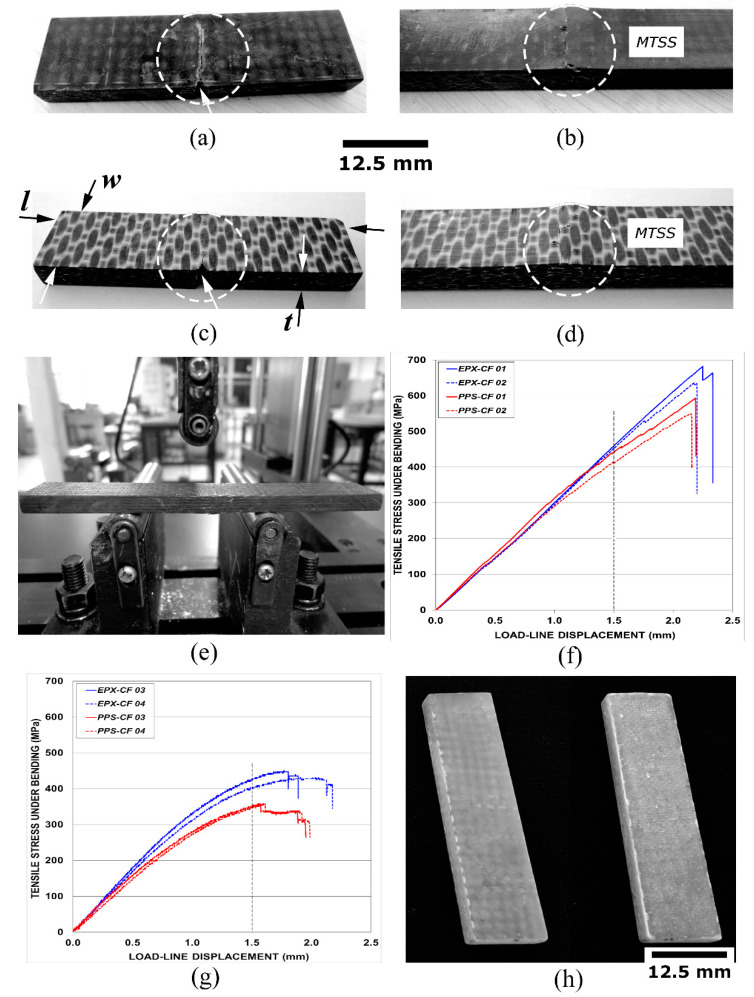
(**a**,**b**) Translaminarly cracked and delaminated carbon fibre-reinforced epoxy resin (EPX-CF) test coupons; (**c**,**d**) and the same for the carbon fibre-reinforced thermoplastic poly-phenylene sulphide (PPS-CF) specimens; (**e**) a three-point bending (3PB) setup supporting a delaminated specimen; (**f**,**g**) flexural loading plots for, respectively, delaminated and translaminarly damaged EPX-CF and PPS-CF test pieces; and (**h**) undamaged, but defective glass fibre-reinforced thermoset epoxy resin (EPX-GF) sample (front and back). Damaged regions are highlighted by dashed circles in (**a**–**d**). Respective surfaces experiencing maximum tensile stress (MTSS) are indicated in (**b**,**d**).

Several full-thickness (t) test coupons in the form of 12.5 mm-width (w) × 50 mm-length (l) tablets were extracted via water-cooled circular diamond saw from the practically void-free (i.e., free from typical manufacturing defects) high-quality EPX-CF and PPS-CF laminates. For both laminates, two test pieces for each fracture mode, namely, translaminar and delamination, were then tested in flexure until their maximum load-carrying capacity, so that the corresponding average fracture strength could be determined. These specimens are shown in Figure 1a,c (translaminar fracture mode) and Figure 1b,d (delamination mode).

Additional specimens (one for each loading mode) were then loaded till a fraction of the respective mean ultimate stress aiming to reproduce the damages present in still-resistant laminates, i.e., exhibiting substantial residual strength. A 1.2 mm-deep notch was machined in the translaminar specimens (arrowed in Figure 1a,c) to favour subsequent fracture by flexural loading (Figure 1e). Tests were carried out under displacement control at a speed rate of 1 mm/min, until their load-line deflection attained 1.5 mm (Figure 1f,g).

According to Figure 1f, at the 1.5 mm deflection the average tensile stress attained approximately 420 MPa in the delaminated PPS-CF specimen’s surface, while nearly 450 MPa was reached for the EPX-CF test coupons. These values correspond to, respectively, 75% and 80% of the estimated peak stress acting in the femur neck in a typical sideways fall-on-the-hip position event [43]. This indicates that a composite orthopaedic implanted device might be damaged in daily events though the corresponding bone structure remains intact. Therefore, deliberately created delaminations in this study are representative of those possibly generated in composite-made hip prostheses during their lifetime and therefore, are worthy of MRI examination towards the structural integrity assessment of implanted devices.

Regarding the translaminarly damaged specimens (Figure 1g), the machined notch clearly diminishes their flexural strength compared to respective delaminated coupons. Besides, one can observe that the stress concentrator (invariably present in structural components) makes the test coupons substantially more compliant. Therefore, at 1.5 mm deflection, the estimated average tensile stress developed on the specimen surface was almost 350 MPa for the PPS-CF, and roughly 410 MPa for the EPX-CF laminate; these figures correspond to, respectively, 60% and 70% of the peak stress related to the hip crash event. This implies a significantly greater likelihood of implant damage without a fracture risk of the femur, thus emphasizing the need for a reliable non-destructive inspection technique for load-bearing composite devices operating in the body´s internal environment.

### 2.3. Defectively Manufactured Test Coupons

Regarding the EPX-GF test coupon (front and back sides shown in Figure 1h), no mechanical damage was imposed to it, since it already contained worthy-of-assessment manufacturing defects (i.e., voids) by MRI technique. It should be emphasized that voids correspond to the fabrication-induced porosity, essentially formed due to the unfilled space of the preform during the impregnation of the fibres.

## 3. MRI Equipment and Protocols

MRI has been successfully applied to imaging the soft tissues of human body, as they are essentially formed by water, a hydrogen-rich compound. Hydrogen is the highest sensitiveness chemical element to the magnetic resonance effect, which refers to the interaction between an externally applied magnetic field and the atomic nuclei having a non-zero magnetic moment. Having just one proton, hydrogen presents a net spin and thus an associated collinear magnetic moment. This causes its nucleus to behave like a little magnet, with the angular momentum vector precessing around its effective magnetic field axis (Larmor precession), which is a fundamental condition for the development of the magnetic resonance phenomenon [25]. In magnetic resonance examination, the inspected article is subjected to the external action of a strong and continuously active static magnetic field, as provided by a superconducting magnet. The magnetic moments related to the nuclear spin of the target object tend to align in parallel or anti-parallel to the static magnetic field. When submitted to a second external magnetic field, oscillating at the radiofrequency portion of the electromagnetic spectrum, the spins absorb energy when hit and excite a resonant frequency. The resulting precession movement originates from the radiofrequency field, and a nearby coil can detect the magnetization precession, whose amplitude will decay over time and a spin-echo signal can be generated (at echo-time) if a rephasing condition is met, such as a refocusing radiofrequency pulse. The reception, decoding and processing of this signal enables the formation of magnetic resonance images [21].

Custom-made horizontal 2.0 Tesla superconducting magnet (Oxford Instruments model 85310HR™), operating at 85 MHz, with 310 mm of internal diameter and equipped with a set of high-speed X, Y and Z gradient coils originally designed to examine small rodents, was employed. Gradient coils operated with an efficiency of 0.16 Gauss/(cm.Ampere), producing magnetic field gradients of about 30 Gauss/cm in a settling time of 160 microseconds. The MRI system run with Bruker Biospin™ electronics and Paravision™ V console, and the following protocols for image generation were utilized: Two-dimensional Rapid Image Acquisition with Refocused Echoes (2D-RARE) [44], and Two-dimensional Fast Low-Angle Shot (2D-FLASH) [45].

Both protocols enabled the acquisition of two-dimensional images composed of pixels. The third dimension was explored by stacking parallel slices, with a slice direction field of view (FOV) determined by the interslice distance and number of slices, which were controlled by the operator.

Basically, RARE is a rapid imaging technique that speeds up image acquisition by acquiring more than one k-space (the matrix representing spatial frequencies in resonance imaging) line per repetition (the number of times that each excitation step is reproduced). The RARE-type sequence comprises multiple π pulses to create many echoes. By applying a different phase encoding gradient to each echo, multiple k-space lines can be collected in each excitation step. The speed-up factor is often referred to as the echo train length, or the turbo factor.

Essentially, FLASH is the first version of a large family of fast gradient-echo methods. It is based on the application of reduced flip angles for magnetic resonance excitation, the acquisition of magnetic field gradient echoes, and considerably shortened repetition times. It is one instance of the generic form of steady-state free precession imaging.

Slice thickness from 1 to 2 mm and proper interslice distance were employed to resolve most of the nuances and details of the inspected composite specimens [37]. The field of view (FOV) was determined by the two dimensions defining the imaging plane, with lower FOV values corresponding to higher digital image resolution and longer image acquisition time. Spatial resolutions utilized during MRI scans ranged from 0.8 × 0.3 mm^2^/pixel (minimum resolution) up to 0.06 × 0.03 mm^2^/pixel (maximum).

**Figure 2:**
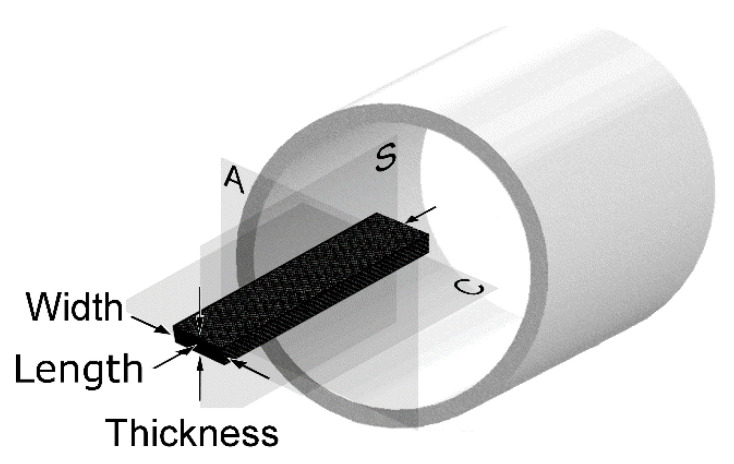
Magnetic resonance imaging (MRI) coordinate system comprising 3 mains view planes: Axial (A) or through-the-specimen length (l), Coronal (C) or through-the-specimen thickness (t), and Sagittal (S) or through-the-specimen width (w).

The MRI coordinate system consisting of Axial (A), Coronal (C) and Sagittal (S) view planes, as related to the test coupon dimensions, is depicted in Figure 2.

## 4. Experimental Procedures

### 4.1. Electrical Conductivity Measurements

Radiofrequency eddy-currents induced in electrically conductive implants might cause significant image artefacts in the MRI experiments [46], so that the electrical properties of inspected materials must be determined. HP milliohmmeter model 4328a™ was used to determine whether the three composite laminates were prone to this detrimental effect. The electrical conductivity of dry pristine specimens was measured according to the four-point probe method [47]. Probes were located 5 mm away from each other. Resistivity was calculated using electrical resistance data, specimen dimensions and geometry, and further converted in electrical conductivity value.

The electrical conductivity of the water-based saline solution (WSS) formulated to simulate biofluids [48], in which the composite specimens were immersed before and during the MRI inspection, was measured through a liquid electrical conductivity meter BSR model COOL EC 302™.

### 4.2. Void and Fibre Contents

The physical and microstructural features of the materials determine their behaviour when subjected to electromagnetic fields, therefore they must be identified and quantified.

While the carbon fibre laminates were manufactured at the industrial scale, following stringent internal quality control standards and procedures to attain the intended fibre-to-resin ratios, respectively, 60:40 (EPX-CF) and 50:50 (PPS-CF), the EPX-GF composite was produced at the laboratory scale, so that its void and fibre content had to be measured. For this purpose, the samples were prepared according to the usual procedures of grinding and polishing with silicon carbide grit and alumina powder, and the three main observation planes were examined in OMAX 40X-2000X Lab LED™ binocular microscope with built-in 1.3 Mp camera. Open source ImageJ™ software (https://imagej.nih.gov/ij/download.html) for digital image analysis was used to remove the noise and process binary images to quantify the pores and fibres.

### 4.3. Immersion in Proton-Rich Liquid Environment

Since the studied materials were inspected under in vitro conditions, their physical interactions with the medium must be established.

Damaged EPX-CF and PPS-CF specimens, along with defective EPX-GF samples (refer to Figure 1), were immersed in WSS at 40 °C, and the respective absorption data points were obtained for the laminates.

After 2.5 months (75 days) of immersion, which is a time-limit beyond which the patient who has been subjected to large-implant surgery can fully resume daily activities [49], purposely damaged EPX-CF and PPS-CF test coupons, along with defective EPX-GF samples, were retrieved towards non-destructive evaluation. They were promptly transferred to falcon tubes filled with WSS, and immediately subjected to MRI examination.

### 4.4. MRI Scanning

#### 4.4.1. Electromagnetic Interference (EMI)

**Figure 3:**
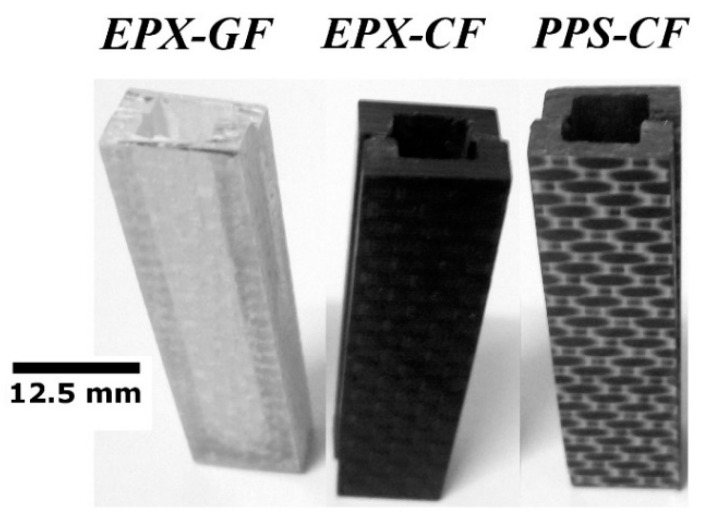
Mini vessels utilized for the detection of MRI artefacts in fibre-reinforced polymer (FRP) laminates. From left to right: EPX-GF, EPX-CF, and PPS-CF composites.

Preliminary MRI tests were carried out aiming at determining the potential EMI shielding due to, respectively, the high electrically conductive nature of carbon fibres and the dielectric behaviour of polymers, which would possibly cause image artefacts. For this purpose, precisely machined U-shaped beams were obtained from EPX-CF, PPS-CF and EPX-GF specimens and glued together to form small-scale containers (Figure 3) to be fully immersed in WSS.

The qualitative ranking of signal-to-noise ratios, contrasts and EMI shielding levels was possible due to differences in the electromagnetic behaviours of the laminates. Each pixel of the magnetic resonance image is assigned a level of visual greyness ranging from black to white that represents the number of hydrogen nuclei, where high hydrogen (i.e., high water) concentration appears as brighter regions, and low concentration to darker areas, as provided by the T2-weighted imaging mode utilized in this study, where T2 is the transverse relaxation time (i.e., T2 magnetization signal decay was compiled towards image acquisition). Therefore, the image contrast refers to the difference in signal strength between adjacent but distinct types of substances, namely solid fibre-reinforced polymer composite (lower T2 signal) and liquid water (higher T2, that is slower T2 decay), which tends to concentrate in damaged or defective areas of the solid laminate. In MRI, the signal-to-noise ratio (SNR) depends inversely on the EMI effect, which is, in turn, directly proportional to the electrical conductivity of the inspected material. This way, the higher the conductivity, the higher the background noise and the poorer the image quality is. It should be noticed that the higher the spatial resolution, the longer the image acquisition time. In addition, sensitivity is increased, and image artefacts are more prone to be created. Resolution, scan time, and SNR describe the limiting factors of an MRI scan [50].

#### 4.4.2. Defective and Damaged Specimens

Test specimens individually inserted in 50 mL plastic falcon tubes containing WSS were loaded to the superconducting magnet. According to Figure 2, the longitudinal axes of the rectangular specimens and the cylindrical magnet were aligned in parallel to one another. It was ensured that the two largest (and parallel) faces of the test coupons faced down and upward, respectively.

The 2D-RARE and 2D-FLASH protocols for image generation were used, and preference was given for the best image quality if the time expenditure in both the setup and imaging steps were not compromised.

### 4.5. Stereoscopic Analysis

An AmScope™ SE305R-AZ-E digital stereo microscope was employed to appraise the amount, orientation, positioning and shape, as well as to estimate the size of deliberately introduced damages in the EPX-CF and PPS-CF test coupons later subjected to MRI inspection. Therefore, the accuracy of the non-destructive technique was judge within the limits applicable to the structural integrity assessments of the composite laminates. According to Ibrahim [51], the damage inspection technique must be able to resolve, with high reliability, an anomaly measuring approximately 5% of the component dimension, containing that discontinuity.

### 4.6. WSS Absorption

ImageJ™ software was utilized for digital magnetic resonance data processing, aiming at estimating the maximum amount (vol%) of WWS absorbed by the defective EPX-GF sample during immersion. The results were compared to the estimations of void content in the dry EPX-GF sample as described in Section 4.2.

## 5. Results and Discussion

### 5.1. Electrical Conductivity Measurements

The electrical conductivities of FRP laminates and WSS fluid are listed in Table 1. The carbon fibre-reinforced polymers (CFRP) exhibited typical values of conductive polymer composites, while a representative value for insulators was found for the glass fibre-reinforced polymer (GFRP) [52]. Interestingly, the biofluid conductivity belongs to a range of conductive polymer composites, approaching well the value determined for simulated body fluid by Abdel-Fattah et al. [53].

### 5.2. Absorbed Liquid by Immersion

**Figure 4:**
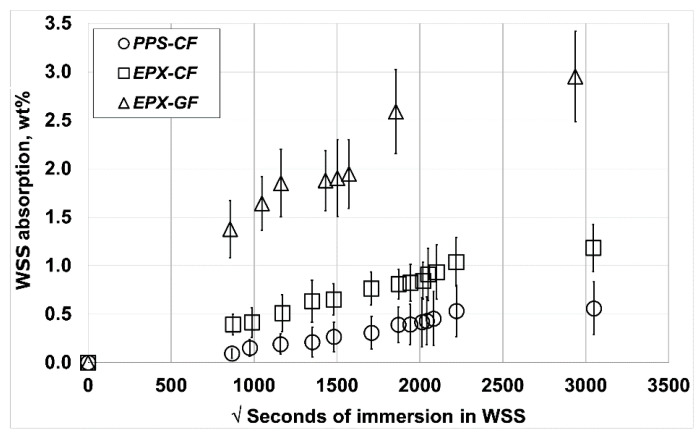
Water uptake plots (wt.%) for the tested laminates immersed in warmed WSS.

Figure 4 displays the water uptake plots (average of three samples) for the three composite laminates tested. The EPX-GF laminate exhibited by far the most hydrophilic behaviour, followed by the EPX-CF and PPS-CF composites, respectively. Sood and Pecht [46] concluded that the water ingress in GFRP mainly occurs through the fibre–matrix interface due to the hydrolysis of the silane glass finish, or from residual thermal stresses. According to the authors, once a path is formed, an aqueous layer can develop through the adsorption, absorption, and capillary action of moisture in that region. Additionally, the high-volume fraction of voids (entrapped air) formed during the GFRP laminate manufacturing at the laboratorial scale must have played an important role, since they accelerated the water intrusion by enlarging the surface area of the polymer matrix exposed to water. Besides, voids possess much higher diffusivity compared to the epoxy matrix, so that enclosed voids act as sites for the storage of water at very higher concentrations, rather than in the resin itself [54].

Regarding the CFRP laminates fabricated at the industrial scale, it can be concluded that the water uptake by the thermoplastic PPS matrix is significantly lower than the thermoset EPX one, which presents similar results to those obtained by [55]. The high crystallinity of the former polymer, along with the low affinity of its chemical structure for water explain this behaviour. Conversely, water absorbed into the EPX resin associates with the secondary hydroxyl polar group, which is the main water absorption centre [56]. The fibre–resin interface of the CFRP laminates is recognizably much stronger and hydrophobic than GFRP, so that water absorption in the former composites is practically restricted to the polymer matrix [57]. According to the Figure 4 data, the minimum volumetric percentage of voids of EPX-CF is around 2.5%, while the PPS-CF approaches 1%. For the laboratorial scale-made EPX-GF, the estimated minimum void content is approximately 6%.

From the data, it can be concluded that a Fickian diffusion linear behaviour with respect to the square-root-of-time is approached at low WSS absorption levels. However, at higher concentrations, a two-stage absorption transient occurs for the thermosetting matrix laminates. This behaviour, which is typical of glassy polymers like epoxy resins, is because diffusion begins to become faster than the polymer chains relaxation [58,59]. While the transition for the EPX-CF laminate occurs after 46 days (2000 squared seconds) of immersion, the EPX-GF exhibits it much earlier, 33 days (1750 squared seconds), which is compatible with the much higher void content presented by the GFRP, as discussed ahead in the text. On the other hand, WSS absorption data for PPS-CF indicate only minor signs of the above cited transition, which is consistent with its partially crystalline structure.

### 5.3. Qualitative and Quantitative Analyses of MRI Data

#### 5.3.1. Electromagnetic Interference (EMI)

**Figure 5:**
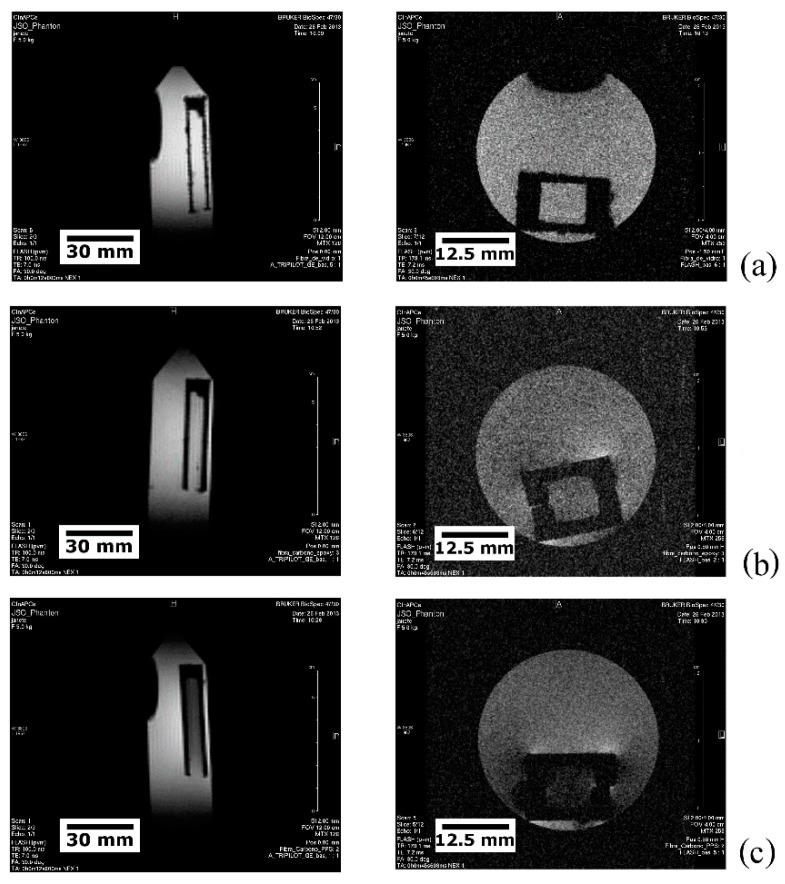
MRI tomographic views of: (**a**) the EPX-GF; (**b**) the EPX-CF; and (**c**) the PPS-CF vessels immersed in WSS.

Figure 5 portrays the longitudinal (left) and transverse (right) tomographic views of the small-scale composite containers (Figure 3) fully immersed in WSS. Scale-bars are provided hereinafter in, respectively, mm and cm to accurately inform the size and dimensions of inspected samples and their discontinuities, like damages and defects.

As seen in Figure 5a, the GFRP vessel presents the lowest EMI shielding (typically not higher than 1 dB in the GHz frequency level) [60], which is supported by the high contrast between the solid (dark) and the liquid (bright, i.e., background signal) phases, as well as by the identical aspect exhibited by the inner and outer fluids. Figure 5b refers to the EPX-CF vial, where moderate EMI noise (typically 40 to 140 dB with a frequency range of 10 Hz to 1.5 GHz) [61,62], causes a somewhat blurred and dull image; besides, image distortion in one of the vessel corners is noticed due to the electromagnetic interference. The PPS-CF container produces the highest EMI signal (Figure 5c) amongst the studied composite laminates, as revealed not only by the low contrast between the inner water and the composite walls, but also from the deviation from the rectilinear projection of the vessel corners.

In conclusion, the synergistically detrimental effect of both the CF reinforcement and the thermoplastic PPS matrix on the MRI inspection is confirmed.

EMI analysis outcomes are, therefore, in full agreement with the electrical conductivity values provided in Section 5.1, i.e., higher conductivities are related to worse imaging in terms of lower phase contrast and signal-to-noise ratio.

#### 5.3.2. Defective and Damaged Specimens

##### Water Absorption Analysis

**Figure 6:**
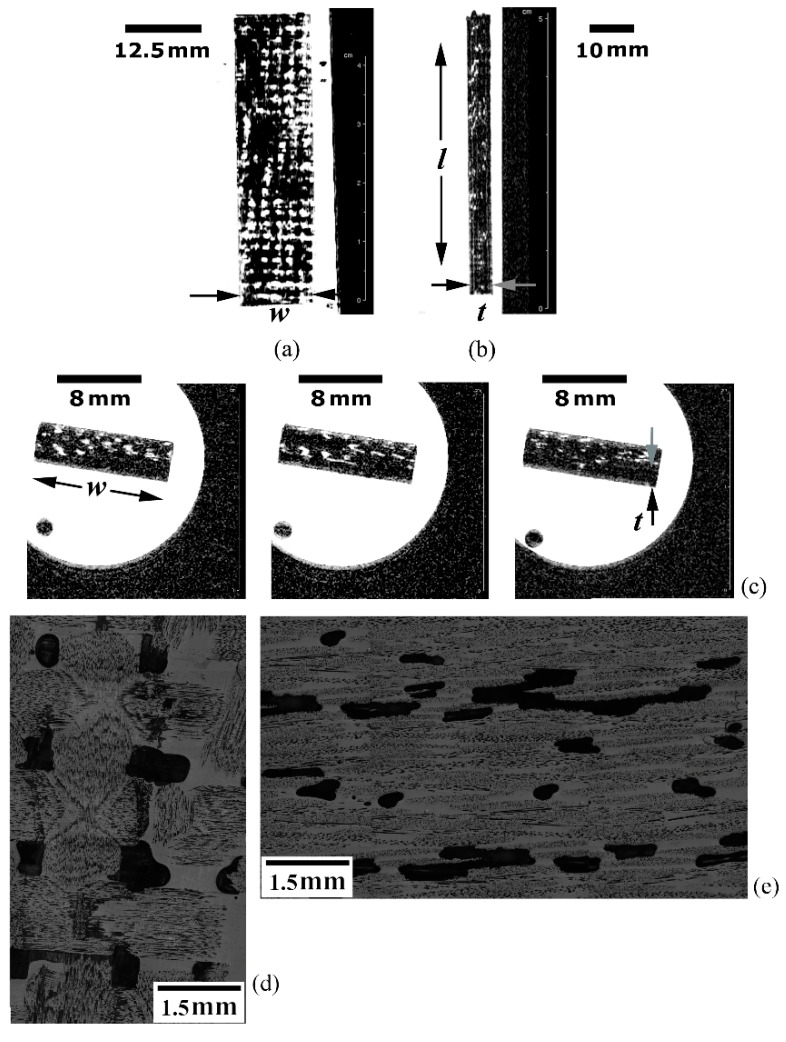
Water absorption analysis of the defective EPX-GF specimen: (**a**) C-view at the specimen mid-thickness; (**b**) S-view at the specimen mid-width; (**c**) parallel slices according to the A-view at the specimen mid-length; and (**d**,**e**) microscopic cross-section views corresponding to, respectively, C and A tomographic planes.

Water absorption analysis is provided in Figure 6a–d, which refer to the defective EPX-GF test coupon. Figure 6a shows a tomographic C-view at the mid-thickness (t/2) position. As intrinsically insulating (Section 5.1, Section 5.3.1), this composite material produces low EMI noise, so that no image distortion occurred. The absorbed WSS (white spots) is confined in evenly distributed cellular compartments, apparently resembling the 0/90 bidirectional plain-weave array of the continuous GF fabric reinforcing the EPX resin matrix.

Figure 6b presents a S-view at the mid-width (w/2) position, where numerous discrete white spots confirm that the EPX-GF laminate absorbs large amounts of liquid, thus corroborating the results supplied in Figure 4. Figure 6c exhibits the tomographic A-views (which are displaced 5 mm relative to each other) at the mid-length (l/2) region of the specimen, where, again, a regular pattern of preferential liquid absorption sites is noticed, though it changes as the cross-section position shifts along the length direction. Less than 35 min were spent acquiring this set of images using the 2D-RARE protocol with l5 repetitions, 27 × 27 mm^2^ FOV, 256 × 256 pixel matrix size (resolution of 0.1 × 0.1 mm^2^/pixel) and 14 ms echo-time.

By following the similar procedure adopted by the authors [37], the estimated value of saline solution absorbed by the EPX-GF sample approached 20%, which is more than three times higher than the value assessed from the water absorption experiments (6%), which can be considered more precise given that the weight measurements were performed in analytical balance and that more than one specimen was monitored over a wide timespan. The more plausible explanation found for this huge difference is related to the image slice-thickness employed in this study, ranging from 1 to 2 mm, so that less than three slices were enough to sample the entire specimen thickness (3.5 mm), with one single slice comprising more than five individual fibre fabric layers of a total of twenty. Therefore, an individual magnetic resonance image (i.e., one single shot) overlapped several laminae and the overestimated water content ensued. This leads to the conclusion that both slice-thickness and interslice distance should, at most, be equal to the layer thickness, which is 180 µm for the tested EPX-GF laminate, to properly determine the water content.

Figure 6d,e provide two different cross-sectioned view planes (respectively, l-w and w-t) of EPX-GF laminate, inspected in reflective optical microscope, where similar discontinuities with respect to those imaged by MRI (Figure 6a–c) can be observed. Because of their high-volume content, the void trend to elongate along the resin flow direction and coalesce before the resin matrix reaches a high enough viscosity to flow [53]. The digital image analysis indicated a void content of approximately 15% in volume, which is not uncommon for the composite laminates manufactured via out-of-autoclave processes [63]. When compared to the 6% of minimum void content estimated from the maximum WSS absorption data point in Figure 4, one can conclude that the original empty space in the defective GFRP laminate were filled not more than a half during the immersion period. This inference agrees with the fact that a plateau has still not been attained for this material until the maximum immersion time displayed in Figure 4.

##### Surface Finish Analysis

**Figure 7:**
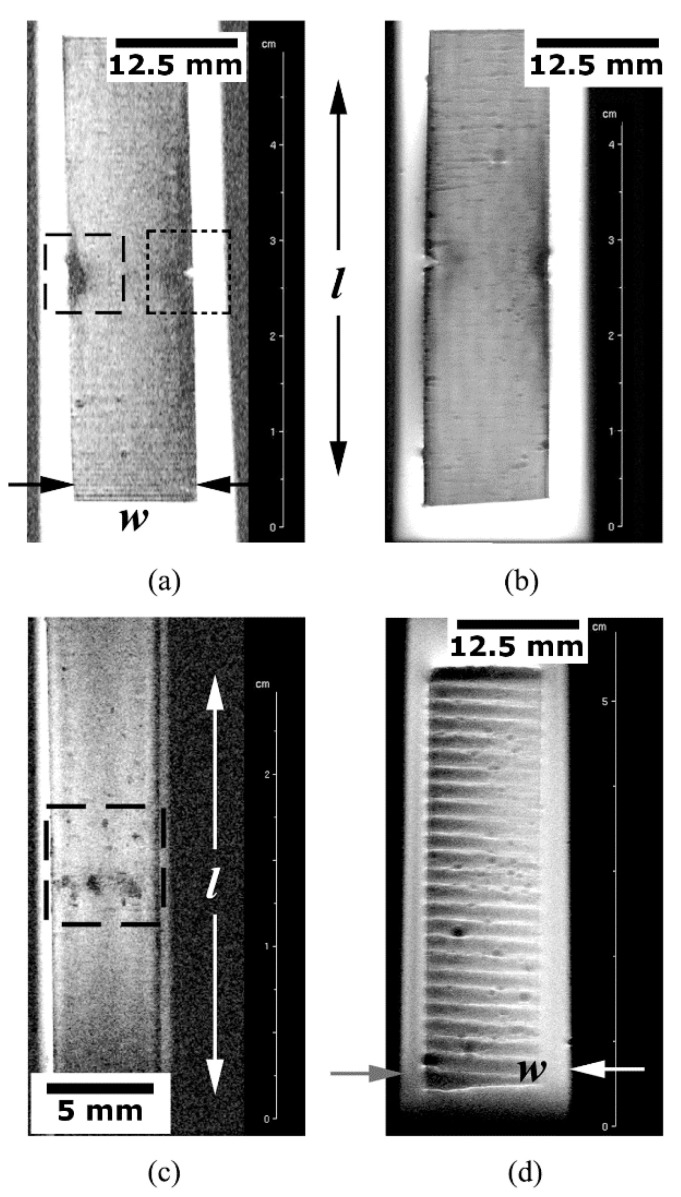
Surface analysis of the translaminarly fractured PPS-CF test coupon: (**a**) C-view with focus set in surface damage and geometrical discontinuity; (**b**) C-view with focus set in surface texture quality; (**c**) S-view of surface crush damage; and (**d**) C-view of the defective EPX-GF surface.

Figure 7a shows the superficial C-view of the PPS-CF test coupon exhibiting a translaminar fracture imaged via the 2D-RARE protocol with seven repetitions, 70 × 30 mm^2^ FOV, 256 × 256 pixel matrix size (resolution of 0.3 × 0.1 mm^2^/pixel) and 14 ms echo-time; the image acquisition process took roughly 40 min. The fracture-induced machined notch is highlighted by the square dotted frame on the right side; to the left, the crush damage caused by the direct contact with the steel-made central loading pin of the three-point bending (3PB) test device is displayed in the square dashed frame. No significative roughness signs are noticed in the examined surface, though some surface waviness is probable due to the fibre fabric array reinforcing the polymer matrix. Evenly spaced horizontal parallel lines on both the top and bottom of the image correspond to artefacts, called “ringing”, which occur near sharp high-contrast boundaries.

Still using the same tomographic C-view, this time applied on the flip side of the same test coupon as before, a higher resolution image was obtained and is shown in Figure 7b. The 2D-RARE protocol was used with 20 repetitions, 70 × 30 mm^2^ FOV, 1024 × 1024 pixel matrix size (resolution of 0.06 × 0.03 mm^2^/pixel) and 14 ms echo-time. The image acquisition time was approximately 160 min aiming to depict the subtle surface features, like the tiny scratches oriented orthogonally to the main specimen axis.

The crush damage shown in Figure 7b is portrayed as the superficial S-view in Figure 7c (square dashed frame). For this purpose, the 2D-RARE protocol was employed at the highest image resolution used in this work, namely, 25 repetitions, 50 × 20 mm^2^ FOV, 1024 × 1024 pixel matrix size (resolution of 0.05 × 0.02 mm^2^/pixel) and 14 ms echo-time, taking the longest acquisition time of almost 200 min, so that even the subtlest surface texture could be fully outlined. This is a vital aspect regarding orthopaedic FRP human implants, since the surface finish ultimately defines the success, or not, of bone tissue-regeneration process [5,9].

In Figure 7d, superficial C view reveals the texture of a defective EPX-GF test piece, were near-surface voids generated by released volatiles during the chemical reaction of bicomponent EPX resin are seen. The 2D-RARE protocol with five repetitions, 70 × 30 mm^2^ FOV, 256 × 256 pixel matrix size (resolution of 0.2 × 0.1 mm^2^/pixel) and 14 ms echo-time; the image acquisition time approached 30 min. Evenly spaced, parallel, and alternated bright–dark steps most probably correspond to artefacts, which may have been aggravated by the large surface roughness exhibited by this poorly manufactured and defective laminate.

Given that typically 40 to 80 min are expended in medical MRI examination [64], one can argue that the images exhibited in Figure 7b,c exceed regular clinical practice. In this regard, it is opportune to recall that an off-the-shelf low-field MRI equipment formerly designed for small rodents’ examination was employed in this study (Section 3). Besides, it must be considered that huge developments have recently been made to significantly reduce the image acquisition time and decrease the susceptibility to motion-related artefacts. For instance, the compressed sensing method [65,66] minimizes the amount of data that is collected for successfully reconstructing magnetic resonance images. Moreover, novel magnetic metamaterials have offered marked enhancements in the signal-to-noise ratio, the image resolution and scan efficiency [67,68]. All together, these improvements are predicted to cut the current examination times by more than a half, which is a good reason to stay optimistic about the future of MRI as a powerful non-destructive assessment tool for composite orthopedic implants.

##### Translaminar Fracture Analysis

**Figure 8:**
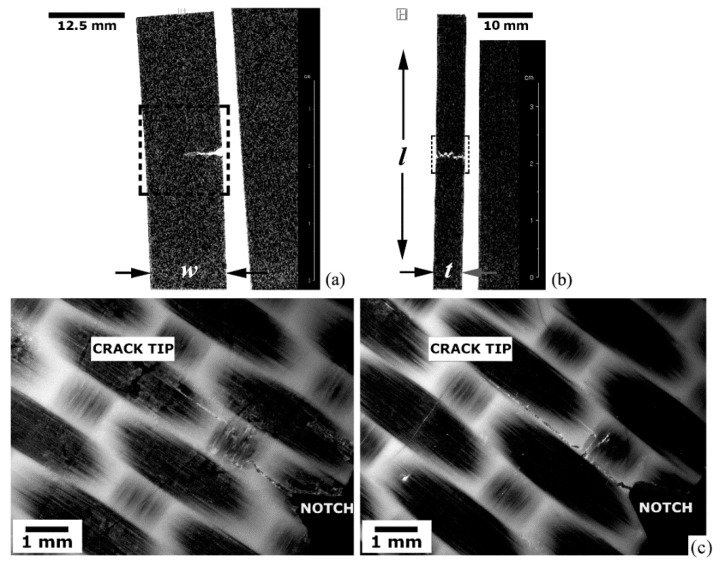
Translaminarly fractured PPS-CF specimen: (**a**) tomographic C-view at mid-thickness of the test coupon; (**b**) S-view at 3.5 one-fourth width from the notched edge of the specimen; and (**c**) stereoscopic images of both l-w surfaces of the specimen, where both the notch and crack tip positions are indicated.

Translaminar fracture analysis of the PPS-CF laminate is presented in Figure 8a–c. Figure 8a portrays the crack path (square dashed frame) according to a tomographic C-view at the mid-thickness of the specimen. The 2D-RARE protocol with five repetitions, 50 × 40 mm^2^ FOV, 312 × 312 pixel matrix size (resolution of 0.16 × 0.13 mm^2^/pixel) was utilized to enhance the scanning speed (total scan time around 20 min) while still maintaining an appropriate spatial resolution. A through-the-width image-slice (S-view) captured at approximately one-quarter (w/4) from the notched-side of the laminate is shown in Figure 8b (small square dotted frame). At that position, the crack opening displacement is still distinctly detectable by MRI, revealing that an equalized crack front progression ensued. Figure 8c provides stereoscopic images of both l-w surfaces of the test piece, where the crack starting-point at the machined V-notch tip, and the final crack tip position are indicated. The crack lengths measured in one and other surfaces of the specimen are a little larger than 6 mm (notch-depth included), indicating a very uniform crack propagation front along the specimen thickness. This value agrees very well with that obtained via MRI at half-specimen thickness (Figure 8a), which corresponds exactly to the half-width of the specimen, i.e., 6.25 mm. Therefore, the differences between real and measured crack lengths do not exceed 4%, which largely satisfies the minimum limit of accuracy in crack size estimation for reliable structural integrity assessments [51], around 0.65 mm for this case study.

##### Delamination Fracture Analysis

**Figure 9:**
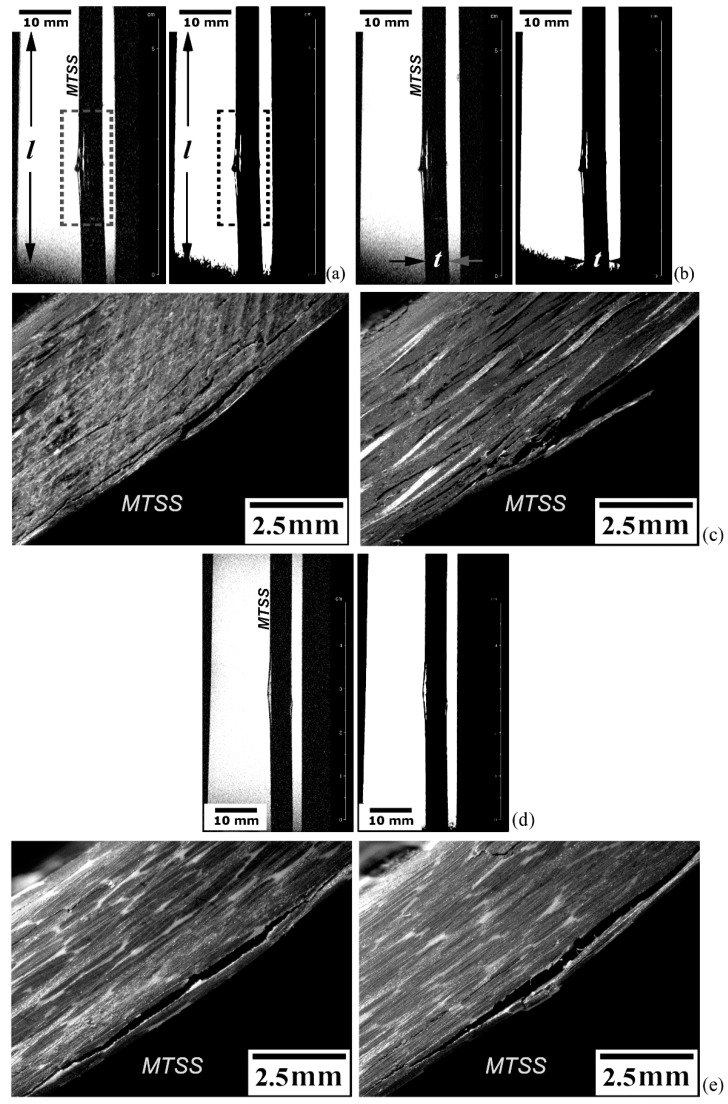
Delaminated FRP specimens: (**a**) S-view (respectively, original and after digital processing images) at the mid-width of EPX-CF via the 2D-RARE protocol; (**b**) via the 2D-FLASH protocol; (**c**) stereoscopic views of both l-t surfaces of the specimen; (**d**) S-view (original and after digital processing images, respectively) at the mid-width of PPS-CF laminate; and (**e**) stereoscopic views of both l-t surfaces of the specimen. MTSS is indicated.

The delamination fracture analysis is exhibited in Figure 9a–e. Figure 9a refers to a tomographic S-view at the mid-width of the EPX-CF laminate presenting interply delaminations (rectangular dotted frame) in the central portion of the inspected test coupon. Exhibited on the left side is the original MRI scan image obtained via the 2D-RARE protocol with eight repetitions, 160 × 40 mm^2^ FOV, 312 × 312 pixel matrix size (resolution of 0.5 × 0.13 mm^2^/pixel) and 11 ms echo-time; 25 min were spent for the entire imaging process. At the right side, the same picture is shown after filtering to improve the signal-to-noise ratio and thresholding to generate a binary image representation. It can be concluded that, while the first image mode reveals the tiniest possible delamination to be detected under specific experimental conditions, therefore providing delamination counting (totalling three interply cracks), the second mode is better in delineating more precisely the contours of the two major (more critical) damages. Therefore, these approaches complement each other regarding to a full depiction of materials discontinuities typically developed in composite laminates, so that their physical features can be more accurately estimated in both qualitative and quantitative terms.

Figure 9b differs from Figure 9a, in that the 2D-FLASH protocol was used towards a shorter exposure time (18 min) by applying 10 replications, 160 × 40 mm^2^ FOV, 192 × 128 pixel matrix size (resolution of 0.8 × 0.3 mm^2^/pixel) and 5 ms echo-time. A faster scan rate implied some quality loss of the original magnetic resonance image, which directly reflected on the same proportion to the respective binary image. As can be noticed as well, the delamination length summation, roughly 1.5 mm for slower MRI scanning (representing less than 3% of the full specimen length), is somewhat higher than the faster scanning protocol, so that the structural integrity assessment would be less conservative in the latter case, which means a riskier approach. Therefore, although timesaving is naturally quite compelling, caution must be taken when selecting proper imaging protocol sequences to prevent missing important material discontinuities and/or misinterpreting them, notably in terms of their size, geometry, number and spatial orientation, since they are primary determinants of the successful practical and theoretical failure analyses of structural composite laminates [69,70].

Figure 9c presents the stereoscopic views of both l-t surfaces of the specimen. While the longest delaminations tend to concentrate next to the surface subjected to the tensile peak stress (maximum tensile stress—MTSS), since transversal shear stresses attain their maximum value in this location, tiny closed delaminations can be seen throughout the specimen thickness, some of them still detectable prior to the original image processing, as shown on left side of Figure 9a,b. Besides the very small opening displacement of tiny delaminations, another reason why these discontinuities were not imaged by MRI is that they might not have crossed the inspected sagittal MRI slice, which was positioned exactly at the central position along the specimen width. As interply damages tend to start at the lateral surfaces of plates subjected to static loading [71], they are likely to be detected as long as slices next to the specimen borders are selected, which is not the case here.

Figure 9d corresponds to a tomographic S-view of the thermoplastic PPS-CF laminate, which is accompanied by the respective binary image representation. The same operational parameters as for the thermoset EPX-CF laminate in Figure 9a were utilized, and a good image quality was also obtained, indicating that the 2D-RARE protocol is suitable for the delamination-damage inspection in both classes of polymer matrix composites. By comparing Figure 9a with Figure 9d, one can notice that the EPX-CF composite is much more delaminated than the PPS-CF material. In fact, the latter composite exhibits just one large interply separation (15 mm long and maximum crack opening displacement of 0.85 mm). This is confirmed by the stereoscopic image provided in Figure 9e, which also shows very few sparse barely visible delaminations. Except for the large shear stress-induced delamination in one specimen surface, and for an unexpected small flexural buckling delamination induced at the opposite surface by the contacting central loading pin, MRI inspection did not detect any other damages, probably due to the same reasons discussed above for the EPX-CF laminate.

Recalling that the PPS-CF laminate is substantially tougher than EPX-CF (Section 2.1), most part of the deformation energy is spent on expenses of adjacent plies separation [72], not to mention that the former material contains much less individual plies per thickness unit, which means fewer potential delamination sites. Besides, it can be also argued that stronger EMI effects by the PPS-CF laminate (Section 5.3.1), along with an insufficient proton-rich liquid ingress in minor (less critical) delaminations due to the intrinsically low potential of this composite to absorb water (Section 5.2), additionally contributed to attenuate the damage detection capability compared to the EPX-CF composite.

It can be concluded from the above that the thermoplastic matrix composite may require more careful consideration regarding MRI inspection, though much probably, according to the results presented herein, most critical damages and defects in this type of material can be promptly effectively detected, identified and characterized by MRI.

##### Analysis of Asymmetrical Translaminar Fracture

**Figure 10:**
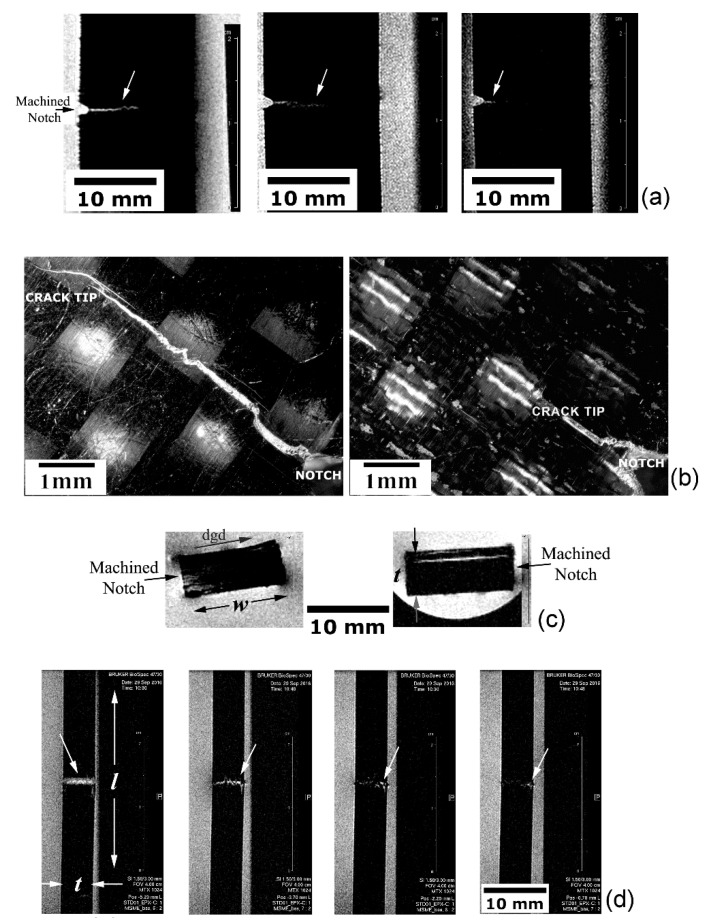
Translaminarly fractured EPX-CF specimen presenting an asymmetrical crack length profile: (**a**) from the longest to the shortest crack length across the specimen thickness; (**b**) the stereoscopic views of the crack at the specimen surfaces; and (**c**) the different crack growth profiles related, respectively, to the translaminar and delamination fractures detected in, respectively, EPX-CF and PPS-CF specimens. (**d**) S-view images at four different through-the-width positions in the EPX-CF coupon, which correspond respectively to the notch tip, 15%, 30% and 45% of w.

Figure 10a–d present the unbalanced or non-uniform translaminar fracture developed in the EPX-CF test coupon. The same operational parameters utilized for this thermoset matrix laminate in Figure 9a apply hereinafter, except for the 80 × 40 mm^2^ FOV leading to a larger image resolution of 0.25 × 0.13 mm^2^/pixel, implying a longer time expenditure of 35 min. Figure 10a refers to the C-view slices at three different through-the-thickness positions, where dissimilar crack lengths are seen. This crack length pattern is confirmed in Figure 10b, where stereoscopic views of both faces of the specimen show, respectively, the longest (6.5 mm) and the shortest (2.5 mm) cracks (notch-depth included).

The left picture of Figure 10c discloses the A-view at the specimen mid-length (l/2) position, i.e., corresponding exactly to the crack propagation plane, where the notch location and the damage growth direction (dgd) are indicated. The brighter region (comprising low grey-level pixels) next to the notch tip corresponds to higher number of hydrogen nuclei, i.e., a WSS-rich area, evidencing that the liquid phase did not spread uniformly throughout the test piece thickness due to the acute crack tip opening gradient. This is in full agreement with the crack front profiles shown in Figure 10a, where the largest crack opening displacement (related to the longest crack) in one flank of the specimen favoured local water ingress, thus providing high responsive magnetic resonance signals; in the opposite flank, however, the smallest crack opening displacement (shortest crack) prevented water access, therefore reducing magnetic resonance signalling.

Just for comparison purposes, the right picture of Figure 10c depicts a peculiar case for a PPS-CF test coupon where, instead of an expected translaminar crack growth, a large delamination was created. Hence, local water ingress allowed the clear interply damage outline by MRI as a bright area.

Figure 10d shows distinct damage states captured by tomographic S-views, which developed in the wake of the crack progression due to the increasing applied load to the EPX-CF test coupon. Towards that end, four parallel slices were imaged, respectively, at the notch tip, and just about 0.7, 2.5 and 4.5 mm ahead of it. The right-hand side crack leading edge (indicated by white arrows) is always discernible, whereas the left-hand crack forefront trends to fade away, thus corroborating the above findings.

It can be inferred from Figure 10 that unveiling more complex translaminar fracture patterns requires all primary MRI view-planes to fully determine the crack location, orientation, size, and shape. On the other hand, the vast majority of delaminations, as assessed in Section 5.3.2, need only one C-view slice to be completely defined.

## 6. Conclusions

This study evaluated the potential of multi-slice magnetic resonance imaging for the in-vitro non-destructive inspection of defective and purposely damaged glass and carbon fibre-reinforced thermoplastic and thermoset polymer matrix laminates immersed in water-based saline solution simulating biofluids.

The main conclusions are as follows:

- The orientation, positioning, shape, and particularly the size of translaminar and delamination fractures were determined according to rigorous structural integrity assessment criteria. For example, the crack-like damage length was measured within 5% of accuracy regarding the most representative specimen dimension.
- The spatial distribution, shape, and contours of the water-filled voids were satisfactory delineated to estimate the amount of absorbed water if thinner imaging slices than this study are used.
- The surface texture of composite specimens featuring roughness, waviness, indentation, crushing, and scratches was fully outlined, with occasional artefacts not prejudicing image quality and interpretation.
- The 2D-RARE image acquisition protocol was judged preferable than the 2D-FLASH, since the latter algorithm favoured non-conservative damage assessment.
- Low electromagnetic shielding glass fibres rendered the highest, while electrically conductive carbon fibre provide the poorest quality images, particularly when the latter reinforcing fibres were mixed to thermoplastic polymer, though trustworthy image interpretation was still attainable.
- The obtained results indicate that magnetic resonance imaging is potentially useful to provide crucial information about flaws´ severity and criticality in fibre-reinforced polymer composite orthopaedic implanted devices for decision-making process, with potential benefits for the patient´s well-being, time and cost-savings when clinical interventions are considered unnecessary on the basis of fracture mechanics approach.

## Figures and Tables

**Table 1:** Electrical conductivity of the FRP composites and the liquid water-based saline solution (WSS) in which they were immersed.

Material	Electrical Conductivity, ohm^−1^·m^−1^
EPX-CF	1.7 × 10^3^
PPS-CF	3.0 × 10^3^
EPX-GF	1.0 × 10^−10^
WSS	28

## Nomenclature

A	Axial
C	Coronal
CF(RP)	Carbon fibre (-reinforced polymer)
dgd	Damage growth direction
EMI	Electromagnetic interference
EPX-GF	Epoxy resin-glass fibre
EPX-CF	Epoxy resin-carbon fibre
FLASH	Fast low angle shot
FOV	Field of view
FRP	Fibre-reinforced polymer
GF(RP)	Glass fibre (-reinforced polymer)
l	Specimen length
MRI	Magnetic resonance imaging
PPS-CF	Poly-phenylene sulphide-carbon fibre
RARE	Rapid image acquisition with refocused echoes
S	Sagittal
t	Specimen thickness
MTSF	Maximum tensile stress surface
w	Specimen width
WSS	Water-based saline solution
2D	Two-dimensional
3PB	Three-point bending
